# Extensive extramedullary haematopoiesis in a patient with non‐transfusion‐dependent beta‐thalassaemia

**DOI:** 10.1111/bjh.20169

**Published:** 2025-06-15

**Authors:** Laura Distelmaier, Wolfgang G. Kunz, Sebastian Theurich

**Affiliations:** ^1^ Department of Medicine III, LMU University Hospital Ludwig‐Maximilians‐Universität München Munich Germany; ^2^ Department of Radiology LMU University Hospital Munich Munich Germany; ^3^ German Cancer Consortium (DKTK), Munich Site, and German Cancer Research Center Heidelberg Germany; ^4^ Comprehensive Cancer Center Munich, CCCM LMU University Hospital Munich Munich Germany; ^5^ Bavarian Cancer Research Center (BZKF) LMU University Hospital Munich Germany

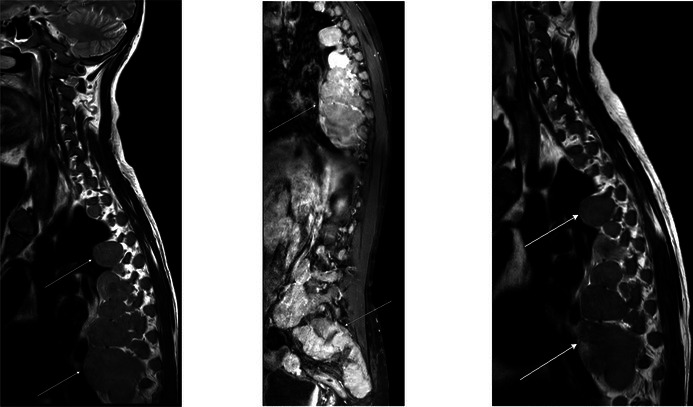

A 52‐year‐old woman with Haemoglobin E/beta‐thalassaemia, which was defined as having non‐transfusion‐dependent β‐thalassaemia (NTDT), a term used for patients with clinically relevant thalassaemia who do not require regular blood transfusion, was referred to us. In her youth, she had been splenectomised because of splenomegaly and low haemoglobin levels. During her 40s, she experienced an aggravation of anaemia with haemoglobin levels around 50–60 g/L, and she then also developed pulmonary hypertension and received home care oxygen and sildenafil treatment. In this situation, regular red blood transfusions were indicated but hindered by the presence of alloantibodies, leaving her with best supportive care but a severely reduced quality of life.

When the patient was referred to us, we suggested a treatment with the Smad2/3 agent luspatercept which is approved for beta‐thalassaemias who are categorised as NTDT or transfusion‐dependent‐thalassaemia.

Since luspatercept treatment can lead to increased extramedullary haematopoiesis (EMH),^1^ we performed an Magnetic Resonance Imaging (MRI) scan of the spine before treatment initiation. The MRI revealed multiple paravertebral soft tissue masses along the whole spine, which were diagnosed as EMH (MRI images left and middle panels, white arrows). Biopsy was not performed because of the typical MRI findings of multiple, symmetric, well‐demarcated, T2w‐slightly hyperintense soft tissue masses with the pathognomonic distribution pattern of EMH along the paravertebral space. Possible differential diagnoses of these manifestation comprise of atypical lymphoma manifestations or extramedullary multiple myeloma, but these were considered unlikely.

Three weeks after luspatercept treatment initiation, haemoglobin levels raised profoundly and stabilised at levels of 90–100 g/L. The treatment was tolerated well and clinical symptoms improved profoundly. Follow‐up of MRI scans during the first year of luspatercept treatment did not reveal further growth of the EMH manifestations (MRI image right panel, white arrows), and thus, specific treatments for progressive EMH, such as radiation or hydroxycarbamide, were not administered.

EMH is a well‐known phenomenon in thalassaemia,^2^ but was especially impressive in this case, most likely due to long‐lasting, low baseline haemoglobin levels of 50–60 g/L.

This case illustrates that, even in asymptomatic NTDT patients, EMH manifestations can be extensive. Due to its mechanism of action, luspatercept can induce further EMH tissue growth and consecutive compression symptoms.^3^ Therefore, MRI scans should be recommended before and during treatment with luspatercept. In case of growing and symptomatic EMH manifestations, termination of treatment and cytoreductive therapies such as irradiation or chemotherapy with hydroxyurea can be necessary.

## PATIENT CONSENT STATEMENT

The patient gave her informed consent to the use of her medical charts, MRI imaging, histology imaging and photos for medical research purpose.
